# The protective role of lidocaine in surgeries involving trigeminal nerve manipulation: a meta-analysis of trigeminocardiac reflex prevention

**DOI:** 10.1007/s10143-025-03449-6

**Published:** 2025-03-20

**Authors:** Almonzer Al-Qiami, Sarah Amro, Khalid Sarhan, Yusra Arafeh, Mina Milad, Islam Omar, Abdulqadir J. Nashwan

**Affiliations:** 1 Faculty of Medicine and Health Sciences, Kassala University, Kassala, Sudan; 2 Specialized Arab Hospital, Rafidia, Palestine; 3 Faculty of Medicine, Mansoura University, Mansoura, Egypt; 4 Jordan University of Science and Technology, Amman, Jordan; 5 Faculty of Medicine, Cairo University, Cairo, Egypt; 6 Faculty of Pharmacy, South Valley University, Qena, Egypt; 7 Medical Research Group of Egypt, Negida Academy, Arlington, MA Egypt; 8 Nursing & Midwifery Research Department (NMRD), Hamad Medical Corporation, Doha, Qatar; 9 Department of Public Health, College of Health Sciences, QU Health, Qatar University, Doha, Qatar

**Keywords:** Lidocaine, Meta-analysis, Trigeminocardiac reflex

## Abstract

The trigeminocardiac reflex (TCR) is activated when the trigeminal nerve is manipulated through mechanical pressure, traction, or irritation. This leads to a rapid increase in parasympathetic activity, resulting in a decrease in heart rate (HR) and blood pressure, which can potentially lead to bradycardia, and cardiac arrest, with a prevalence of 14.5% during neurointerventional procedures (8). The aim of this study was to assess the effect of lidocaine injection on preventing TCR during cranial surgeries. We conducted a comprehensive search of PubMed, Web of Science, and Cochrane CENTRAL electronic databases for clinical trials comparing the use of lidocaine versus placebo, or no intervention for TCR prevention during cranial surgeries up until 20 May 2024. Meta-analyses were performed using fixed-effects models, and heterogeneity was assessed using I-squared and Chi-square tests. A total of five studies involving 421 patients were included in this meta-analysis. The incidence of TCR was significantly lower in the lidocaine group compared to the control group, with a risk ratio of 0.05 (95% CI 0.01 to 0.37, *P* = 0.003). Additionally, the lidocaine group had a relatively small change in HR, with a mean difference of -10.56 (95% CI -13.30 to -7.83 beat per minute, *P* = 0.00001). No statistically significant difference in mean arterial pressure was observed between the two groups (MD -5.15, 95% CI [-10.38 to 0.08 mmHg], *P* = 0.09). Our results suggest that lidocaine may be effective in preventing TCR and stabilizing HR. The use of lidocaine could be considered a prophylactic measure during cranial surgeries. Further studies are needed to investigate the optimal dose and timing of lidocaine administration.

**Supplementary Information:**

The online version contains supplementary material available at 10.1007/s10143-025-03449-6.

## Introduction

The trigeminocardiac reflex (TCR) is a relatively unknown reflex. It is considered a subtype of trigemino-vagal reflexes and occurs when the trigeminal nerve is manipulated anywhere along its course either by mechanical pressure, traction, or irritation. When triggered, it leads to rapid parasympathetic activity, decreasing HR and blood pressure [1].

It occurs more commonly during surgical interventions that involve manipulation of the trigeminal nerve, such as neurosurgery, maxillofacial surgeries, and ENT surgeries [2–7], with a prevalence of 14.5% during neurointerventional procedures [8]. However, it can be triggered by non-surgical interventions or manipulations, including non-invasive procedures like dental work or nose and eye examinations that involve contact with the trigeminal nerve [9, 10]. Kratschmer observed TCR for the first time in 1870 [11], while the establishment of the peripheral variant of the TCR, the oculocardiac reflex, was accomplished by Aschner and Dagnini in 1908 [12].

TCR is classified into two subtypes: central TCR, which is activated by stimulation of the intracranial portion of the trigeminal nerve from the Gasserian ganglion to the brainstem, and peripheral TCR, which is triggered by stimulation of the trigeminal nerve at any point outside the skull up to the Gasserian ganglion [13].

It is essential for surgeons to possess knowledge about TCR and the importance of its prevention. The initial and foremost management option for TCR is to minimize any mechanical trauma that may be inflicted upon the nerve along its course. It is essential to take into account the risk of developing TCR when conducting any procedures that are carried out in the course of the trigeminal nerve. The use of pharmacological treatment to prevent TCR is still a controversial topic and requires more research to prove its efficacy and safety. Atropine, an anticholinergic drug, has been proposed in several studies as a possible potential pretreatment to prevent severe bradycardia in TCR [14–16]. Despite the reduction in the rate of bradycardia, high doses of atropine injection were not sufficient to provide complete protection against TCR [17]. The use of lidocaine and local anesthetics has been proposed as a novel strategy to prevent TCR in several studies [17–20]. Lidocaine blocks sodium channels, inhibiting nerve impulse transmission and reducing sensory input from the trigeminal nerve to the brainstem, where TCR is initiated [21, 22] . However, a study showed that the injection of lidocaine and adrenaline combination in the left lateral osteotomy sites in rhinoplasty procedures did not prevent TCR and most cases resolved spontaneously after the stoppage of surgical stimulus [23].

Nevertheless, the knowledge regarding the utilization of lidocaine in TCR is still limited, and there are many unanswered questions regarding its safety, efficacy, and optimal dosage. These unresolved questions need to be well addressed aiming to decide whether implicating it will be effective in clinical practice or not. Therefore, we conducted a systematic review and meta-analysis of the existing clinical trials that involve the usage of lidocaine in the prevention of TCR. Our primary objective was to assess the efficacy of lidocaine injection as a potential prophylactic treatment for TCR.

## Methods

The protocol of the present systematic review has been registered to PROSPERO (ID: **CRD42023426950**). We adhered to Preferred Reporting Items for Systematic Reviews and Meta-Analysis (PRISMA) guidelines during all stages of this review.

### Eligibility criteria

We included all studies that met the following criteria: 1. Randomized, controlled trials (RCTs) that compared lidocaine with placebo or any other drug; 2. Studies whose population included participants of all ages who were undergoing cranial surgeries; 3. Studies with lidocaine intervention; 4. Studies with control groups taking any medication other than Lidocaine to prevent TCR; 5. Studies reported at least one of the following variables: Incidence of TCR, defined as the abrupt occurrence of bradycardia induced by surgical manipulation of the trigeminal nerve and its associated anatomical divisions, changes in heart rate (HR) the bradycardia was identified by a decrease in heart rate of at least 20% from the initial level, and change in mean arterial pressure (MAP). Studies that were not available in the English language were excluded due to potential translation errors and incomplete data, thesis, conference abstracts, and studies with unreliable data for extraction and analysis had been excluded.

### Literature search strategy

In order to conduct a comprehensive search, we utilized various databases including PubMed, Web of Science, and Cochrane CENTRAL. To ensure thoroughness, we also made use of the MESH database and specific search terms as follows:

((lidocaine) OR (lignocaine) OR (xylocaine) OR ("local anesthetic")) AND ((trigeminocardiac) OR (asystole) OR (“sudden arrhythmia”) OR ("Parasympathetic dysrhythmia") OR (“sudden bradycardia”) OR (“sudden Hypotension”) OR.

(“hypotension shock”) OR (“sympathetic hypotension”)) AND ((Endovascular) OR

(Embolization) OR (maxillofacial) OR (intracranial) OR (ophthalmic) OR (dental))

The search was conducted from the inception of the databases from 12 to 20th May 2024, Two authors screened the titles and abstracts of the retrieved citations.

Disagreements were resolved by discussion. Eligibility screening was performed in two steps; the first step was to screen titles and abstracts for eligibility. In the second step, full-text articles of eligible abstracts were retrieved and screened for eligibility for meta-analysis.

### Data extraction

Two independent authors extracted the data from an online data extraction sheet. The extracted data included: (1) study design; (2) study population; (3) type of surgery; (4) lidocaine dose; (5) method of administration; (6) side effects; (7) risk of bias domains; and (8) study outcomes: Incidence of TCR, baseline HR, and MAP, HR, and MAP after the manipulation assumed to cause TCR. Data was rechecked by a third reviewer, who also resolved any conflicts between reviewers.

### Quality assessment

To assess the quality of included RCTs, the Cochrane Handbook for Systematic Reviews of Interventions 5.1.0 (updated March 2011) was utilized. For this purpose, we used the quality assessment table provided in the previously mentioned book. The Cochrane risk of bias assessment tool includes various domains such as sequence generation (selection bias), allocation sequence concealment (selection bias), blinding of participants and personnel (performance bias), blinding of outcome assessment (detection bias), incomplete outcome data (attrition bias), selective outcome reporting (reporting bias), and other potential sources of bias. The authors' judgment was classified as 'Low risk,' 'High risk,' or 'Unclear risk' of bias.

### Dealing with missing data

When graphs were the only means of presenting the mean and/or standard deviation around it for the baseline or manipulation point, we used a graph reader online site [24] to obtain these values. If authors only reported diastolic and systolic pressure and their respective means instead of MAP, the formula:

MAP = ((Systolic pressure) + (2 × Diastolic pressure))3 was employed to calculate MAP, while the Cochrane formula: Pooled SD = (SD1)2 + (SD2)2 was used to calculate the standard deviation around MAP means.

### Data synthesis

Changes in HR and MAP were pooled as mean difference (MD) in the meta-analysis model, while the Incidence of TCR was pooled as Risk Ratio (RR) in the fixed effect model using the Mantel–Haenszel method. We used Review Manager 5.4 for Windows.

### Sensitivity analysis

We conducted a sensitivity analysis to address the heterogeneity if present and to make sure that none of the included studies affected the results, and to examine whether the overall effect size was statistically reliable (see “Results”).

### Assessment of heterogeneity

We first evaluated heterogeneity through forest plots visual inspection, followed by measurement using the I-square and Chi-square tests. The Chi-square test was utilized to determine the presence of significant heterogeneity, while the I-square test quantified the magnitude of heterogeneity in effect size. We interpreted and assessed heterogeneity based on recommendations outlined in Chapter 9 of the Cochrane Handbook of Systematic Reviews and Meta-analysis, a Chi-square test alpha level below 0.1 was considered significant heterogeneity, while I-square values were interpreted as follows:

0–40% may not be important, 30–60% may represent moderate heterogeneity, and 50–90% may represent substantial heterogeneity. If significant heterogeneity was present, a random effects model was used; otherwise, a fixed effect model was employed.

## Results

**Fig. 1:**
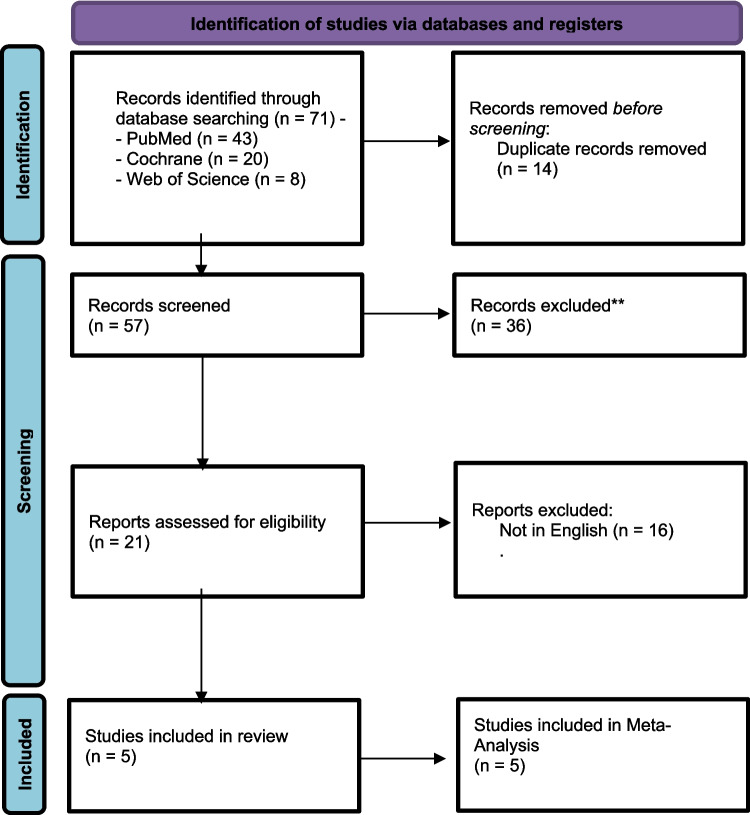
PRISMA flow diagram of studies’ screening and selection

Our search retrieved 71 articles. Following the abstract screening, only 21 titles were eligible for full-text screening. Finally, five RCTs [19, 20, 23, 25, 26] with a total of 421 patients were found to be eligible for the final analysis (see PRISMA flow diagram; Fig. 1).The included studies were Zhang et al. (2022), Bohluli et al. (2011), Yorgancilar et al. (2012), Sun et al. (2023), and Tibano et al. (2010). (19–21,24,26)Out of the five studies that were included, two focused on patients who underwent percutaneous balloon compression (PBC) [19, 26] Another study involved patients who underwent bilateral sagittal split ramus osteotomy [20], a third study examined patients who underwent rhinoplasty [23] and the fourth study by Sun et al. focused on patients who received onyx embolization during cerebrovascular intervention surgery [25] The largest dose was 40 mg [23] while the smallest one was 10 mg [19]. Lidocaine was administered intravenously in three studies [26] [25] [19] and as a nerve block in two studies [20, 23]. The summary of the included studies and their main results are shown in Table 1 supplementary file No. 1, and the baseline characteristics of their populations are shown in Table 2 supplementary file No. 1.

### Quality of included studies:

**Fig. 2:**
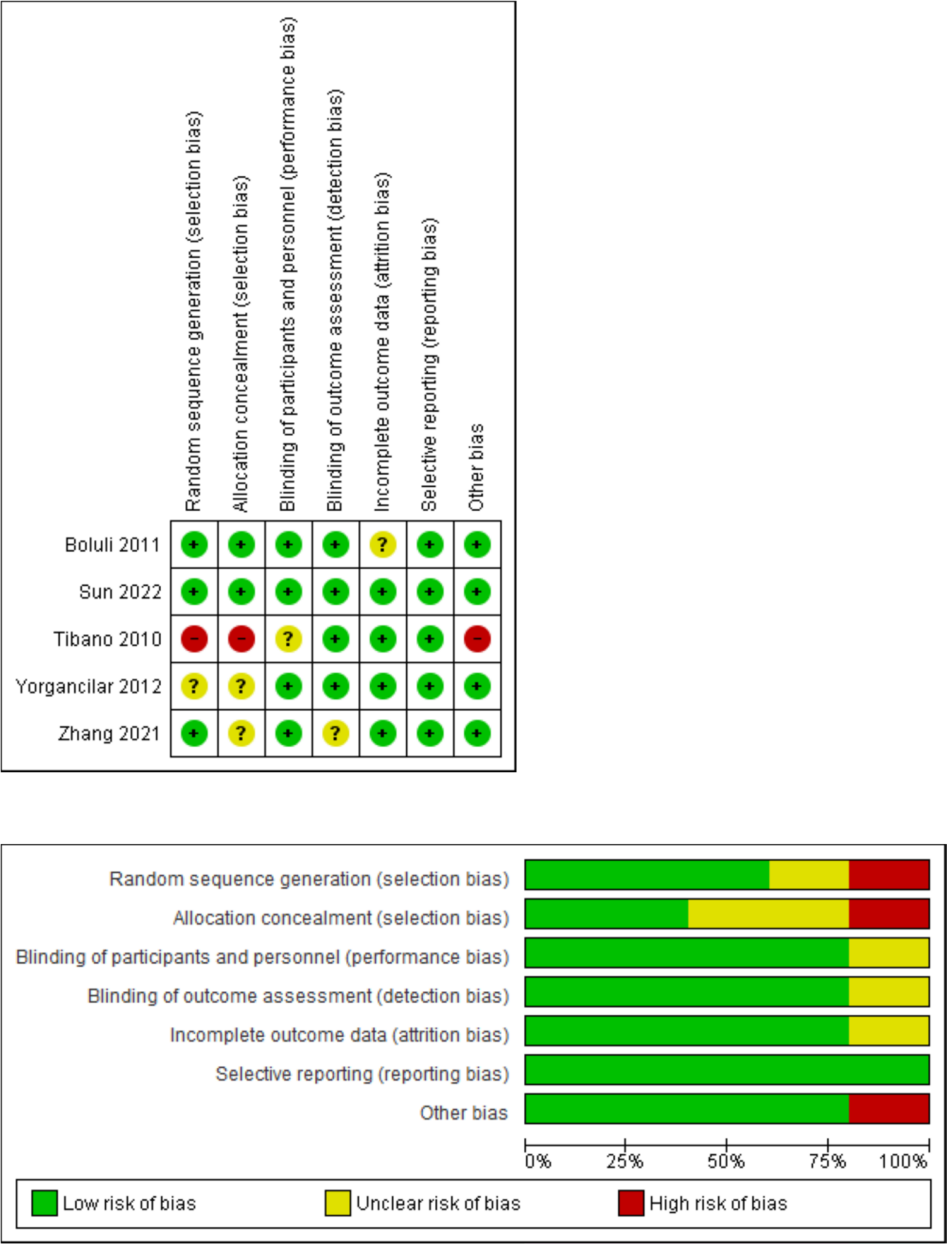
The risk of bias summary and risk of bias graph according to the Cochrane risk of bias assessment tool

The quality of the included studies ranged from moderate to high quality according to the Cochrane risk of bias assessment tool. The summary of quality assessment domains of included studies is shown and authors’ judgments with justifications in Fig. 2.

### Efficacy analysis (from baseline to the time of manipulation)

1. **Incidence of TCR.**

The overall risk ratio favored lidocaine over the control drug (RR 0.25, 95% CI

**Fig. 3:**
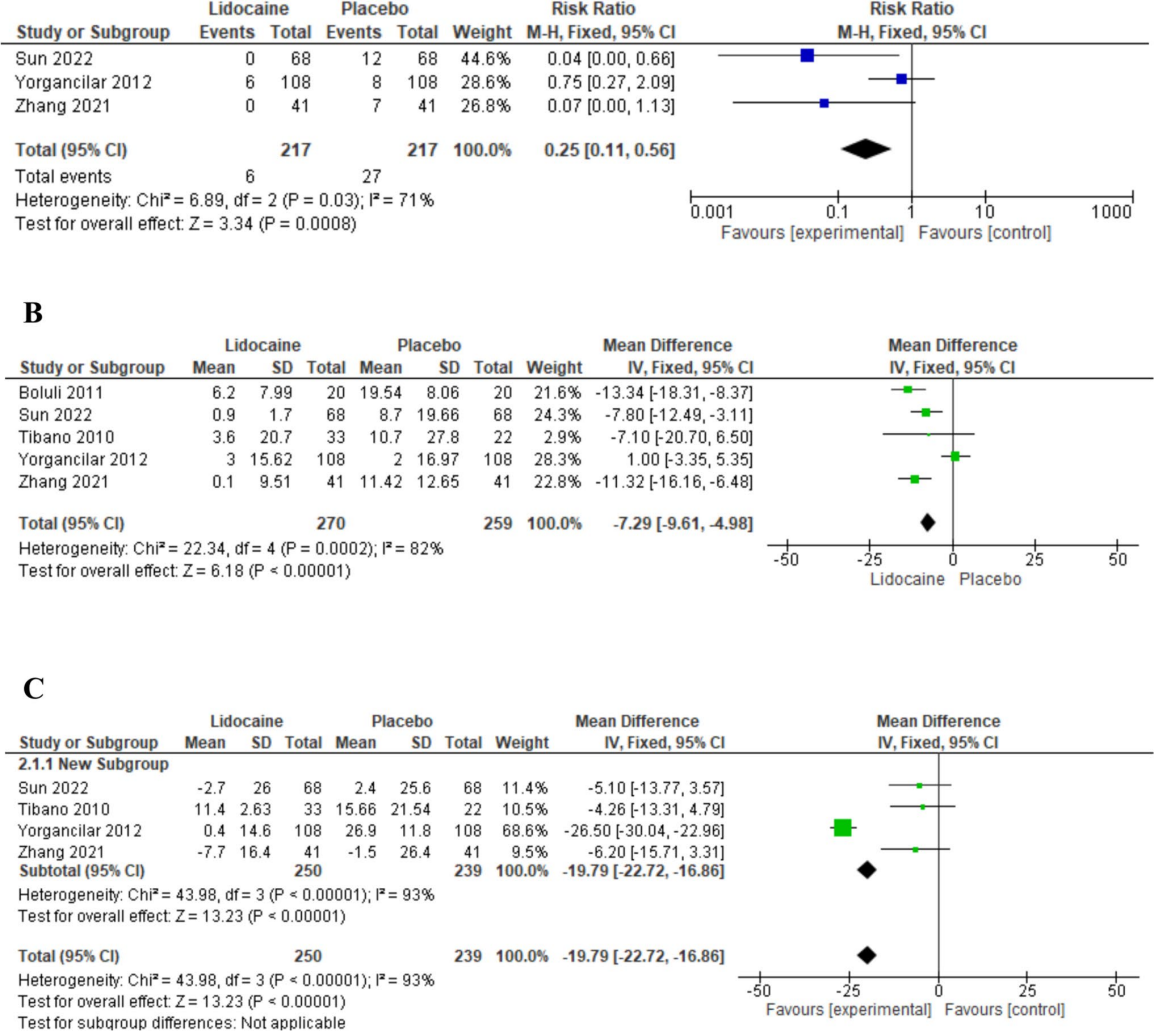
Forest plot of the change in efficacy measures from baseline to manipulation point **a**) Risk Ratio of the change in Incidence of TCR. **b**) Mean difference of the change in Heart rate. **c**) Mean difference of the change in mean arterial pressure MD mean difference, IV inverse variance, CI confidence interval

[0.11, .56], P = 0.0008). . Pooled studies demonstrated substantial heterogeneity (*P* = 0.03, I-square=71% Fig. 3a). Sensitivity analysis was conducted in which one study was excluded in each scenario. The best resolution of heterogeneity was achieved by excluding the study of Yorgancilar et al. 2012 [23] (*P* = 0.80, *I*-square=0%).

**Fig. 4:**
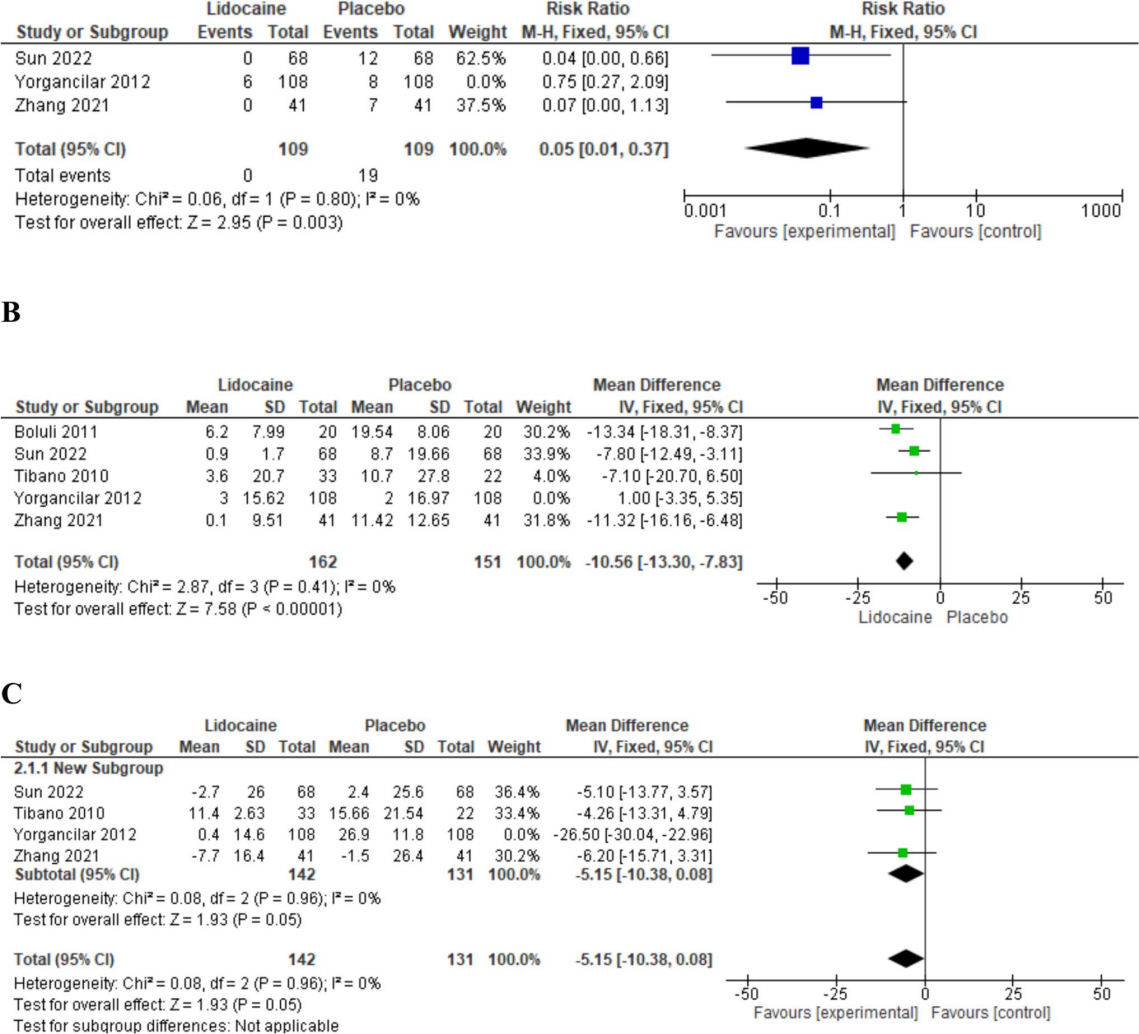
After sensitivity analysis Forest plot of the change in efficacy measures from baseline to manipulation point **a**) Risk Ratio of the change in Incidence of TCR. **b**) Mean difference of the change in Heart rate. **c**) Mean difference of the change in mean arterial pressure) MD mean difference, IV inverse variance, CI confidence interval

After this exclusion from the meta-analysis model, the overall Risk Ratio favors Lidocaine drug over the control drug (RR 0.05, 95% CI 0.01 to 0.37, *P* = 0.003;

Fig. 4a). All studies except for Yorgancilar et al. were RCTs whereas Yorgancilar

2. **Change in heart rate**

The analysis results favored lidocaine over the Control drug (MD −7.29, 95% CI [−9.61 to −4.98 bpm], *P* = 0.00001; Fig. 3b). However, there was substantial heterogeneity between the pooled studies (*P* = 0.0002, *I*-square=82%). To address this, sensitivity analysis was conducted, excluding one study at a time. The best resolution of heterogeneity was achieved by omitting the study of Yorgancilar 2012 [23] After removing this study from the meta-analysis model (*P* = 0.41, *I*-square=0%), the overall mean difference is still in favor of the lidocaine drug over the control drug (MD −10.56, 95% CI [−13.30 to −7.83 bpm], *P* = 0.00001; Fig. 4b). A dose-effect relationship was observed across the included studies, with higher doses of lidocaine generally associated with greater reductions in HR. For a detailed discussion of the dose-effect dynamics, including the influence of surgical type and TCR risk, see the Discussion section.

3. **Change in mean arterial pressure**

The overall mean difference favored the Lidocaine drug over the control drug (MD −19.79, 95% CI [−22.72, −16.86 mmHg], *P* = 0.00001; Fig 3c). Pooled studies were not homogenous (*P* = 0.00001, *I*-square=93%). To resolve the heterogeneity, we conducted a sensitivity analysis in multiple scenarios, excluding one study in each scenario. Heterogeneity was best resolved by excluding the study of Yorgancilar et al. 2012 [23] After removing it from the meta-analysis model (*P* = 0.96, *I*-square=0%), the overall mean difference did not favor the drug lidocaine over the drug placebo (MD −5.15, 95% CI [−10.38 to 0.08 mmHg], *P* = 0.09; Fig 4c).

### Sensitivity analysis

Except for the analysis of the MAP variable, where the study Yorgancilar 2012 [23] may contribute to its superiority (see “Discussion”), lidocaine remains superior in all efficacy outcomes. The observed differences in efficacy outcomes could be attributed to the heterogeneity observed when this study was included. The uniqueness of this study, as it was the only one that resolved heterogeneity when excluded from analysis, suggests that this was not a chance occurrence. Furthermore, this pattern was observed across all variables analyzed, not just one. Despite similar participant demographics among all studies, there may have been underlying characteristics or comorbidities that were not considered in the analysis of Yorgancilar's study. Additionally, the distinct clinical condition and the different study design employed by Yorgancilar et al. further justify the decision to exclude it from our analysis.

## Discussion

The incidence of TCR was lower in the lidocaine group compared to the control group, with a risk ratio of 0.05 and a 95% confidence interval of 0.01 to 0.37. The heterogeneity observed in our analysis may stem from differences in surgical types and control group protocols. Intracranial procedures, such as percutaneous balloon compression (PBC) (Zhang et al., 2022; Tibano et al., 2010) and cerebrovascular interventions (Sun et al., 2023), involve direct trigeminal nerve stimulation, which is a strong TCR trigger [19, 25, 26] In contrast, peripheral procedures like bilateral sagittal split ramus osteotomy (Bohluli et al., 2011) and rhinoplasty (Yorgancilar et al., 2012) manipulate the mandibular and maxillary branches, respectively, which may carry a lower TCR risk [20, 23]. This variability in surgical risk likely influenced TCR magnitude and lidocaine's effectiveness, as seen in the smaller MAP reduction in rhinoplasty compared to intracranial procedures [23]. Additionally, inconsistencies in control group protocols may have contributed to heterogeneity. Studies using sham injections or placebo (e.g., Zhang et al. and Yorgancilar et al.) accounted for placebo effects, providing controlled comparisons [19, 23]. In contrast, studies using no intervention (e.g., Bohluli et al. and Tibano et al.) may have overestimated lidocaine's effect due to the absence of placebo controls [20, 26] Furthermore, studies using standard care (e.g., Sun et al.) introduced variability due to differing institutional protocols and potential confounding interventions [25]. These factors likely contributed to the heterogeneity in outcomes such as HR and MAP. When compared to control drugs, patients who received lidocaine injection experienced a relatively minor change in the mean HR with a reduction of 10.56 beats per minute, while the concern of TCR starts when it decreases by more than 20 bpm as suggested in the literature [27]. The MAP was decreased by 5.15 mmHg in the lidocaine group, despite the marginal statistical significance (*P* = 0.05), a change of 5.15 mmHg in MAP is too small to be considered clinically relevant as a TCR. These findings suggest that lidocaine could enhance patients' MAP stability by preventing the occurrence of TCR.

The significant reduction in HR observed in the control groups relative to the lidocaine groups implies that lidocaine exerts an influence on the autonomic nervous system and cardiovascular stability. The effects of lidocaine on cardiac parasympathetic control in normal subjects and in subjects after myocardial infarction [28] The mechanism of lidocaine entails its interaction with sodium channels located within neurocellular membranes, resulting in transient inhibition of sodium influx and impeding membrane depolarization [21, 22]. This effect is also applicable to sensory nerve fibers, which are usually impacted initially due to their slender configuration and heightened vulnerability to penetration [29]. By blocking sodium channels, lidocaine also may inhibit the release of neurotransmitters such as glutamate and substance P from sensory nerve fibers in the trigeminal nerve. This reduces sensory input from the trigeminal nerve to the brainstem, where TCR is initiated [30]. With reduced sensory input, there is less stimulation of the parasympathetic nervous system, which leads to a decrease in HR and blood pressure, this prevents the reflex response associated with TCR [1].

When conducting the sensitivity analysis, we noticed that the study by Yorgancilar [23] had an impact on the overall effect of Lidocaine in terms of its superiority in the analysis of MAP. When excluding Yorgancilar's study, lidocaine was found to be statistically superior to the control drug in terms of MAP change (with a marginal P value = 0.05). However, when considering the remaining variables, Yorgancilar et al.'s study contributed to the inferiority of lidocaine and caused the heterogeneity between studies to be significant. It is important to note that Yorgancilar et al.'s study had the largest number of participants and was the only study with a crossover design, which may explain the significant heterogeneity observed when including it in our analysis. It is also important to mention that MAP can either rise or fall during surgical manipulation, and this change whether it is positive or negative is considered alongside HR as an indicator for the occurrence of TCR.

There are two administration forms, one of them is the "Go Gate block" which refers to the administration of lidocaine typically before the induction of anesthesia. This form of administration aims to provide pain relief and reduce the likelihood of TCR activation during the initial stages of the surgical process. The other one is the "after induction" form of lidocaine administration, which involves delivering lidocaine after the patient has been induced with anesthesia and the surgical procedure has commenced. This method is often used to manage pain and potentially prevent TCR activation during the later stages of the surgery [31]. One aspect of the included studies, by Yorgancilar et al. [23] and Buhloli et al., [20] to be considered is the administration and timing of the trigeminal nerve block. The block was administered after the induction of anesthesia, which made it impossible to confirm the depth and effectiveness of the block in Yorgancilar`s study. However, according to Buhloli et al. study in which they used the Gow-Gate block, it is important to acknowledge that previous research has reported a high success rate (up to 98% in some studies) when using the Gow-Gates block[32]. Additionally, a previously published study suggests that an additional nerve block may inhibit the peripheral aspect of the reflex [20]. However, it cannot completely suppress the central component [33]. In this study, they followed the conventional approach of triggering the peripheral aspect of the reflex and aimed to minimize its impact by utilizing the Gow-Gates block. Nevertheless, it is crucial to remember that a peripheral nerve block alone cannot completely prevent the occurrence of the TCR reflex [34]. It is worth noting another point that Bohluli et al. used pulse rate (PR), instead of HR in their study, which is a less precise measure and quite different from HR. This could be seen as a limitation of our study, as we were unable to use a standardized mean difference in HR analysis due to the expected heterogeneity caused by study design and clinical condition disparities.

The incidence of TCR may be influenced by the degree of manipulation of the trigeminal nerve. Several factors support this claim: Firstly, animal studies have shown that TCR can be elicited by different levels of trigeminal nerve stimulation, with stronger stimulation resulting in more frequent and pronounced reflex responses [35]. Secondly, studies have indicated that TCR is more likely to occur when there is direct mechanical stimulation or manipulation of the trigeminal nerve, particularly its ophthalmic and maxillary divisions. This suggests that a higher degree of manipulation may surpass a certain threshold, leading to an increased incidence of TCR [36, 37]. Thirdly, the complexity of the anatomical connections of the trigeminal nerve means that the intensity of its manipulation may vary depending on individual anatomical variations. Manipulation near the Gasserian ganglion, where the ophthalmic, maxillary, and mandibular divisions converge, may have a greater impact on TCR incidence due to its proximity to vital cardiorespiratory centers [27]. Finally, the sensitivity of mechanoreceptors in the trigeminal nerve may also play a role, as a result, higher degrees of manipulation could lead to increased activation of these mechanoreceptors, triggering a more pronounced reflex response [37]. In neonates and infants, immature metabolic clearance and lower levels of α1-acid glycoprotein (AAG) result in a higher unbound fraction of lidocaine, extended elimination half-life, and increased risk of accumulation, particularly with continuous infusions [38]. For TCR prevention, careful dosing is essential to avoid toxicity. In children under 3 years, no more than 1.2 mL of a 2% lidocaine solution should be applied, with at least a 3-h interval between doses and a maximum of 4 doses within 12 h. Further research is needed to establish safe and effective dosing protocols for TCR prevention in pediatric populations [39]. In older patients, the lowest effective volume and concentration of lidocaine should be used to minimize systemic toxicity while preventing TCR. The recommended concentration is 10 mg/mL, with dosing not exceeding 5 mg/kg. This approach ensures effective suppression of TCR while reducing the risk of complications [40].

The studies we included in our analysis used varying doses of lidocaine, ranging from 10 to 40 mg, and produced different effects on HR and MAP stabilization. The relationship between the dose of lidocaine and its ability to prevent TCR is not straightforward. Some studies suggest that higher doses of lidocaine may be more effective in preventing the reflex, while others have found no significant difference in effectiveness between different doses. To investigate this further, we compared the Mean Differences for HR and MAP of each study to the dose used (see Table 3 supplementary file No. 4). Interestingly, the study by Bohluli et al., which used a higher dose of lidocaine (36 mg), showed a relatively greater change in HR (MD −13.34) compared to other studies with lower doses of lidocaine (MD −11.32 for Zhang et al., MD −7.8 for Sun et al., MD −7.10 for Tibano et al., MD 1 for Yorgancilar et al., and). However, when it comes to changes in MAP, the highest dose (40 mg) by Yorgancilar et al. was associated with the smallest reduction in MAP compared with lower doses (MD −26.5 vs. for −5.1 Sun et al. study, MD −6.2 for Zhang et al.)

We conclude that determining the optimal dose of lidocaine remains unclear and requires further investigation. It is possible that factors such as weight could contribute to its efficacy, as the literature suggests that lidocaine has an excellent safety profile when administered at doses below 4.5 mg/kg, with some recommendations advocating for a dosage of 2 mg/kg(18). The same for age and overall health status which may influence the optimal dosage for individual patients. Therefore, healthcare providers should carefully consider these factors when determining appropriate dosages of lidocaine for their patients.

Most of the studies included in our analysis did not prioritize the evaluation of lidocaine's safety as a primary outcome. However, Bohluli et al. reported that lidocaine did not result in any significant adverse effects or complications among their study participants. Conversely, Sun et al. documented several adverse events, including dizziness, postoperative nausea, vomiting, muscle weakness, and other severe unexpected events in 13 out of 68 patients (19.1%). Yorgancilar et al., Tibiano et al. and Zhang et al., on the other hand, did not explicitly mention any side effects associated with lidocaine administration. It is noteworthy that Sun et al.'s study utilized intra-arterial injection as the route of administration for lidocaine, which may explain why only participants in this study experienced side effects while others did not. That's why, it is essential to discern whether observed adverse events are a consequence of lidocaine specifically, inherent manifestations of the anesthetic procedure, or indicative of the underlying TCR pathology.

Our findings align with numerous studies utilizing lidocaine as an intervention for TCR during surgeries. Several case reports have recommended the use of lidocaine to counter TCR. For instance, Sun et al. [41] documented two cases in which intra-arterial injections of 10–20 mg of lidocaine during endovascular embolization effectively suppressed TCR. Similarly, Coleman et al [18] reported successful termination of TCR by administering intra-arterial lidocaine during the endovascular embolization of a carotid sinus fistula. These reports highlight the effectiveness of lidocaine in managing TCR, despite implementing other measures such as increasing anesthesia levels and utilizing glycopyrrolate which was ineffective, In addition, other studies supported the use of topical lidocaine like Meuwly et al., [42], other studies recommended using lidocaine combined with other medications like anticholinergic or atropine than lidocaine alone. Yoshida et al. [18] and Kim. et al. [43, 44], reported the use of IV lidocaine infiltration, IV atropine, and IV glycopyrrolate respectively as effective measures for TCR inhibition.

However, some studies have produced contradictory results. For example, Cho et al [45] reported a case of TCR in an 18-year-old female who underwent upper lip surgery to remove a hemangioma. Lidocaine was not effective in stopping the TCR event, as this is a case report it is important to consider individual patient factors, some patients may have a higher risk of developing TCR due to underlying conditions “in Cho et al. study, maybe the hemangioma”, or medications they are taking. In cases like these, we suggest considering alternative preventative measures like using atropine, or lidocaine in combination with other medication. In general, stopping surgical manipulation should be considered as the first reaction to TCR, especially in cases with serious hemodynamic instability. Our included study by Bohluli et al. demonstrated that during surgery, ceasing further tissue manipulation for a few seconds was sufficient to manage cases where critical decreases in PR were observed [20]. Another study also demonstrated that most cases resolved spontaneously after the stoppage of surgical stimulus [25]. Similarly, Qin et al. [46] reported a case of TCR that lidocaine failed to suppress during percutaneous balloon compression in a patient with trigeminal neuralgia, While one of our included RCT studies [19], reports the opposite results for the same procedure. Qen et al. recommended Isoproterenol infusion as a measure to suppress TCR. However, it is worth noting that Qin et al. employed a low dose of lidocaine (1 ml of 2% lidocaine, equivalent to 20 mg), which according to the literature may not have been sufficient for the patient's weight (66 kg). Therefore, the use of a low lidocaine dose in Qin et al.'s study might have influenced its effectiveness in suppressing the TCR reflex.

To the best of our knowledge, this is the first meta-analysis to evaluate the effectiveness of lidocaine in suppressing the TCR reflex during cranial surgeries. The search strategy was comprehensive and systematic, covering multiple databases and using sensitive search queries. The quality assessment and data extraction process was rigorous, following the PRISMA guidelines and the Cochrane risk of bias tool. A sensitivity analysis was used to account for heterogeneity and to assess the robustness of the results. Additionally, the results of the present study are consistent with previous individual studies, further supporting their reliability.

Yet, there are some limitations to our study. The number of included trials is small which may limit generalizability. Additionally, because our search retrieved only three parallel trials, we included crossover with parallel RCTs. Also, the trials only focused on the short-term effects of lidocaine as a TCR inhibitor without considering possible postoperative complications or long-term outcomes. Lastly, the included RCTs only represent specific types of surgeries and may not apply to other ones.

## Recommendation

Further research should focus on TCR in a broader range of surgeries by conducting additional trials to further explore the effectiveness of lidocaine. Investigating the impact of lidocaine on other relevant physiological parameters, such as oxygen saturation or end-tidal carbon dioxide levels, could provide a more comprehensive understanding of its potential benefit. In addition, investigating post-operative complications and long-term outcomes of using lidocaine against TCR will help to build a strong clinical indication and guide its practice. Further studies are needed to explore dose–response relationships of lidocaine for TCR prevention, including comparisons of different doses and administration timings to determine the most effective and safe protocol and correlate all this to the type of surgeries.

## Conclusion

We conclude that lidocaine may be effective in preventing TCR during surgeries where there is manipulation of trigeminal nerve or its branches as it can stabilize the HR and mean blood pressure. The use of lidocaine could be considered a prophylactic measure for TCR. Further studies are needed to investigate the optimal dose and timing of lidocaine administration for TCR prevention during this type of surgeries.

## Supplementary Information

Below is the link to the electronic supplementary material.

Supplementary file1 (DOCX 16 KB)

## Data Availability

No datasets were generated or analysed during the current study.
