# Supplementary material for: The protective role of lidocaine in surgeries involving trigeminal nerve manipulation: a meta-analysis of trigeminocardiac reflex prevention
Source: Neurosurg Rev. 2025 Mar 20;48(1):310. doi: 10.1007/s10143-025-03449-6 (PMC11923009; doi:10.1007/s10143-025-03449-6)
Supplement: Supplementary file 1 — Supplementary file1 (DOCX 16 KB) [file 10143_2025_3449_MOESM1_ESM.docx]

| Study ID | Study Design | Study Population | Sample size | Lidocaine Dose(mg) | Primary and Secondary Outcomes | Results |
| --- | --- | --- | --- | --- | --- | --- |
| Bohluli 2011 | Double-blind (split-mouth) RCT | Patients undergoing bilateral sagittal split ramus osteotomy | 20 | 36 | The primary outcome appears to be the effect of the Gow-Gates block on the incidence of the Trigeminocardiac Reflex (TCR) during bilateral sagittal split ramus osteotomy. The researchers measured the pulse rate at various stages of the surgery and compared the results between the blocked and control sides.  The secondary outcome could be the understanding of the complex neurophysiologic mechanism of TCR and the potential prevention of peripheral TCR. | No statistically significant differences were found between the blocked and control sides except during ramus sagittal splitting and setback manipulation (*P < 0.0001*) when a significantly decreased pulse rate was recorded for the control ramus compared with the blocked ramus. |
| Sun 2022 | Double-blind RCT | Patients undergoing Endovascular embolization during cerebrovascular surgeries | 136 | 20 | **Primary Outcomes:**  Heart Rate (HR) and Mean Arterial Pressure (MAP) were closely monitored during the embolization procedures.  The incidence of Trigeminocardiac Reflex (TCR), mainly characterized by a decrease in HR of ≥20%, was recorded.  During dimethyl sulfoxide (DMSO)/Onyx injection, HR was much slower in the control group than in the lidocaine group.  TCR occurred in 12 patients (17.6%) in the control group (cardiac arrest in 3 patients) with decreased (7 cases) or increased (5 cases) MAP.  No TCR was observed in the lidocaine group.  **Secondary Outcomes:**  Most TCR episodes occurred in patients with dural arteriovenous fistula and the middle meningeal artery being affected.  The composite adverse events were significantly higher in the control group than in the lidocaine group. | During dimethyl sulfoxide (DMSO)/Onyx injection, HR was much slower in the control group than in the lidocaine group (*P < 0.05*). TCR occurred in 12 patients (17.6%) in the control group (cardiac arrest in 3 patients) with decreased (7 cases) or increased (5 cases) MAP, whereas no TCR was observed in the lidocaine group. Notably, most TCR episodes occurred in patients with Dural arteriovenous fistula and middle meningeal artery being affected. The composite adverse events were significantly higher in the control group than in the lidocaine group *(P < 0.05)* |
| Yorgancilar 2012 | Prospective crossover clinical trial | Patients undergoing osteotomy during rhinoplasty procedure | 108 | 40 | **Primary Outcomes:**  The study primarily aimed to determine the blood pressure changes and occurrence of the trigeminocardiac reflex (TCR) during rhinoplasty.  The authors found that TCR was detected in nine patients following lateral osteotomies and nasal pyramid infracture procedures (8.3%).  **Secondary Outcomes:**  The authors determined that Lidocaine and adrenaline combination (LAC) injection prior to osteotomy did not prevent TCR.  They also noted that manipulation at or near the infraorbital nerve during rhinoplasty may cause TCR, even if local anesthetic infiltration is used.  This study contributes to the understanding of TCR during rhinoplasty, which has not been sufficiently discussed among otolaryngologists. | TCR was detected in nine patients following lateral osteotomies and nasal pyramid infracture procedures (8.3%). The authors determined that LAC injection prior to osteotomy did not prevent TCR. |
| Zhang 2022 | Double-blind RCT | Elderly patients with trigeminal neuralgia undergoing Percutaneous Balloon Compression | 82 | 10 | **Primary Outcomes:**  Heart Rate (HR):  The HR decreased in the control group at the time of foramen ovale puncture (T5) and at the time of ganglion compression (T6) compared with that at the moment of needle puncture (T4), but almost no change in the study group. The HR was lower in the control group compared with the study group at T5 and T6.  Mean Arterial Pressure (MAP): The MAP increased significantly at T5 and T6 compared with that at T4 in the control group, but almost no increase in the study group. Compared with the control group, MAP was lower at T5 and T6 in the study group.  **Secondary Outcome:**  Incidence of Bradycardia: The incidence of bradycardia was higher in the control group than that in the study group.  The study concluded that trigeminal ganglion block was an effective approach to prevent Trigeminocardiac Reflex (TCR) in elderly patients during Percutaneous Balloon Compression (PBC). | HR decreased in the C group at the time of foramen ovale puncture (T5) and at the time of ganglion compression (T6)  compared with that at the moment of needle puncture (T4) (*P* < 0.05), but almost no change in the S group. HR was lower in the  C group compared with the S group at T5 and T6 (*P* < 0.05). MAP increased significantly at T5 and T6 compared with that at T4 in the C group (*P* < 0.05), but almost no increase in the S group. Compared with the C group, MAP was lower at T5 and T6 in the S group (*P* < 0.05). There were no significant differences in HR and MAP between the two groups at T1, T2, T3, T4, and T7. The incidence of bradycardia was higher in the C group than that in the S group (*P* < 0.05). |

**Table 1** Summary of included studies

| Study ID | Group | Sample size | Age(Years) | Gender Male % | Baseline HR(bpm) | Baseline MAP(mmHg) |
| --- | --- | --- | --- | --- | --- | --- |
| Bohluli 2011 * | Lidocaine | 20 | 19-40 ¥ | 55 | 90±5.9 § | N/R |
|  | Control | 20 | 19-40 | 55 | 90±5.9 | N/R |
| Sun 2022 ¤ | Lidocaine | 68 | 45.9±15.7 | 63.2 | 64.6±7 | 81.7±8.6 |
|  | Control | 68 | 42.2±15.1 | 58.8 | 65.2±7.2 | 80.6±8.4 |
| Yorgancilar  2012 * | Lidocaine | 108 | 25.3±3.8 | 55 | 79±12 | 86.3±17 |
|  | Control | 108 | 25.3±3.8 | 55 | 75±12 | 85.6±18.6 |
| Zhang 2022 ¤ | Lidocaine | 41 | 63.17±8.26 | 43.9 | 65.4±7.3 | 84.8±9 |
|  | Control | 41 | 65.32±9.03 | 53.7 | 65.6±8.1 | 84.1±12.6 |

**Table 2** Baseline characteristics of included studies; N/R Not Reported, ¤ Parallel trial, * Crossover trial, § Mean±SD, ¥ range

| Study ID | DOSE in mg | MD of HR (bpm) | MD of MAP (mmHg) |
| --- | --- | --- | --- |
| Bohluli 2011 | 36 | -13.2 | N/A |
| Sun 202l | 20 | -7.8 | -5.1 |
| Yorgancilar 2012 | 40 | -2 | -26.5 |
| Zhang 2022 | 10 | -11.3 | -6.2 |

**Table 3** Showing MD of HR and MAP in terms of Lidocaine dose; MD Mean Difference, HR Heart Rate, MAP Mean Arterial Pressure.
